# Copy number variants in lipid metabolism genes are associated with gallstones disease in men

**DOI:** 10.1038/s41431-019-0501-7

**Published:** 2019-09-04

**Authors:** Eduardo Pérez-Palma, Bernabé I. Bustos, Dennis Lal, Stephan Buch, Lorena Azocar, Mohammad Reza Toliat, Wolfgang Lieb, Andre Franke, Sebastian Hinz, Greta Burmeister, Witigo von Shönfels, Clemens Schafmayer, Peter Ahnert, Henry Völzke, Uwe Völker, Georg Homuth, Markus M. Lerch, Klaus Puschel, Rodrigo A. Gutiérrez, Jochen Hampe, Peter Nürnberg, Juan Francisco Miquel, Giancarlo V. De Ferrari

**Affiliations:** 1 Institute of Biomedical Sciences, Faculty of Medicine and Faculty of Life Sciences, Universidad Andres Bello, Santiago, Chile; 2 Cologne Center for Genomics (CCG), University of Cologne, Cologne, Germany; 3 Genomic Medicine Institute and Neurological Institute, Cleveland Clinic, Cleveland, OH USA; 4 Epilepsy Center, Neurological Institute, Cleveland Clinic, Cleveland, USA; 5 Psychiatric & Neurodevelopmental Genetics Unit, Broad Institute of MIT and Harvard, Cambridge, MA USA; 6 Department of Medicine I, University Hospital Dresden, Technische Universität Dresden, TU Dresden, Dresden, Germany; 7 Departamento de Gastroenterología, Facultad de Medicina, Pontificia Universidad Católica de Chile, Santiago, Chile; 8 Institute of Epidemiology and Biobank PopGen, Christian-Albrechts-University of Kiel, Kiel, Germany; 9 Institute of Clinical Molecular Biology, Christian-Albrechts-University of Kiel, Kiel, Germany; 10 Department of Visceral and Thoracic Surgery, University Hospital Schleswig Holstein, Kiel, Germany; 11 Institute for Medical Informatics, Statistics, and Epidemiology (IMISE), University of Leipzig, Leipzig, Germany; 12 LIFE – Leipzig Research Center for Civilization Diseases, University of Leipzig, Leipzig, Germany; 13 Institute for Community Medicine, University Medicine Greifswald, Greifswald, Germany; 14 Interfaculty Institute for Genetics and Functional Genomics, University Medicine Greifswald, Greifswald, Germany; 15 Department of Familiar Medicine, Faculty of Medicine, Pontificia Universidad Católica de Chile, Santiago, Chile; 16 Institute for Integrative Biology, FONDAP Center for Genome Regulation, Departamento de Genética Molecular y Microbiología, Pontificia Universidad Católica de Chile, Santiago, Chile

**Keywords:** Structural variation, Cholelithiasis

## Abstract

Gallstones Disease (GSD) is one of the most common digestive diseases requiring hospitalization and surgical procedures in the world. GSD has a high prevalence in populations with European or Amerindian ancestry (10–20%) and the influence of genetic factors is broadly acknowledged. However, known genetic variants do not entirely explain the disease heritability suggesting that additional genetic variants remain to be identified. Here, we examined the association of copy number variants (CNVs) with GSD in a sample of 4778 individuals (1929 GSD cases and 2849 controls) including two European cohorts from Germany (*n* = 3702) and one admixed Latin American cohort from Chile (*n* = 1076). We detected 2936 large and rare CNVs events (size > 100 kb, frequency < 1%). Case-control burden analysis and generalized linear regression models revealed significant association of CNVs with GSD in men, with the strongest effect observed with CNVs overlapping lipid metabolism genes (*p*-value = 6.54 × 10^–4^; OR = 2.76; CI 95% = 1.53–4.89). Our results indicate a clear link between CNVs and GSD in men and provides additional evidence that the genetic components of risk for GSD are complex, can be sex specific and include CNVs affecting genes involved in lipid metabolism.

## Introduction

Gallstones disease (GSD) is a multifactorial chronic metabolic disease characterized by the development of calculi inside the gallbladder composed mainly by cholesterol. GSD features a long silent progression where the accumulation of stones in the affected gallbladder can lead to clinical symptoms such as recurrent episodes of intense abdominal pain (biliary colic) and major complications, like obstruction and infection of the biliary tree (cholestasis and cholangitis), acute pancreatitis, and gallbladder cancer [1]. At least 20% of the affected individuals will reach the symptomatic phase and the only definitive solution constitutes the surgical removal of the gallbladder (i.e., cholecystectomy), a procedure that carries a high economic cost to health systems [1]. Disease prevalence ranges between 10 and 20%; however, this value is highly variable and strongly influenced by sex, age, body mass index (BMI), and ethnicity. Indeed, women are consistently more affected than men and as age increases so does the probability to develop GSD [2]. Ethnically, groups with European and Amerindian ancestry have the highest prevalence, while populations from Africa or Asia exhibit lower incidence and prevalence [1]. Accordingly, European women above 50 years old from Germany show a prevalence of 39% while women of Mapuche–Huilliche Chilean Native American ancestry in the same age groups are reported to have a prevalence of GSD higher than 75% [1, 2].

GSD has a moderate to strong genetic component [2, 3] with an estimated heritability (i.e., the fraction of cases explained by additive genetic factors) above 25% in Europeans [4] and as high as 50% in admixed Latin American populations [2, 5]. To date, the strongest and most consistently replicated common genetic variants associated with GSD reside in the ATP-binding cassette subfamily G member 5/8 *ABCG5/8* gene (rs11887534; hg19, chr2:44066247G>C) [3, 6] and in the UDP glucuronosyltransferase family 1 member A1 *UGT1A1* gene (rs6742078; hg19, chr2:234672639G>T), with the latter significantly associated with GSD only in men [7]. Both variants explain from ~50 down to 29% of the heritability observed for European and Latin populations [5], respectively, which suggests that additional variants remain to be discovered. A recent meta-analysis involving 27,174 GSD cases versus 736,838 controls identified 32 gallstone disease association signals in 29 genes [8]. Although these genes are involved in lipid and glucose metabolism, as well as in inflammatory response processes, which are strongly related to the etiology of the disease [1, 9], the signals reported exhibit modest to low effect sizes (OR < 2) conferring minimal risk to the carriers [10]. Moreover, we have recently reported ethnic-specific genome-wide associations in a high-risk admixed Latin American population with no evident replication of the aforementioned meta-analysis signals [6], reflecting the complex genetic composition of GSD.

Copy number variants (CNVs) are defined as genomic segments between 50 pb and 3 Mb in size that can result in partial loss or gain of chromosomal material, in comparison with a reference genome [11, 12]. Since their discovery, CNVs changed the diploid paradigm of the human genome, being highly represented along its entire sequence and covering more nucleotides than all SNPs together [11]. At the functional level, and depending on their location, CNVs can cause dosage changes that alter gene expression or produce alternative transcripts or induce gene fusion events [13]. The changes caused by CNVs have been linked to disease. For instance, multiple genome-wide association studies (GWAS) have recently focused on the study of the association of these variants with a broad spectrum of complex traits [14], including metabolic phenotypes such as obesity [15] and diabetes [16].

Here, we performed a CNV burden analysis in 1929 GSD cases and 2849 controls in high-risk European and Latin American cohorts. We examined available datasets individually and then performed a linear regression model to extract general conclusions. We further explored disease-specific CNV gene content, CNV behavior (gain vs. loss) and sex-specific effects. Our results show that CNVs-affecting genes involved in lipid metabolism and expressed in the enterohepatic axis are associated with GSD in men.

## Materials and methods

### Study subjects

Initially, a total of 5568 individuals were collected through three independent studies: (i) The PopGen Population Genetics Biobank (POPGEN) from Kiel, Germany, comprising 2274 individuals of European origin; (ii) The Study of Health in Pomerania second follow-up (SHIP-2) from Greifswald, Germany, with 2059 individuals of European origin; and (iii) The Chilean Latin American cohort (ANCORA) from Santiago, Chile, comprising 1235 individuals [6]. POPGEN and SHIP-2 dataset were conceived as population based cohorts designed to study complex and prevalent diseases in German populations, including cardiovascular, neurodegenerative, and metabolic disorders such as GSD. The ANCORA dataset was specifically built for GWAS on GSD and by design is enriched with women to account for genetic signals within this higher risk group. For all samples, the GSD case state was defined by either a prior history of cholecystectomy or the presence of gallstones determined by abdominal ultrasound. All controls included in this study were required to have a confirmed gallstone-free state by ultrasonography. No matching criteria between cases and controls were involved in the study design. Sample age, sex, BMI, and if available Type 2 Diabetes status (T2D) were extracted directly from cohort main database. T2D status for SHIP-2 was not available and total T2D prevalence was extracted from Schipf et al. [17]. For all three datasets, written informed consent was obtained from all participants and the corresponding ethics committees approved study protocols. Further specific details regarding study design and recruitment for POPGEN [18], SHIP-2 [19], and ANCORA [6] datasets are provided in their original publications.

### Genotyping and sample quality controls

POPGEN and ANCORA datasets were genotyped under the AXIOM® genome-wide platform [19] (version EUR 1 with 674,518 markers and LAT 1 with 817,551 markers, respectively) using the GeneTitan® Multi-Channel (MC) Instrument, following the manufacturer’s instructions. The SHIP-2 cohort was genotyped using the Affymetrix Genome-Wide Human SNP Array 6.0 (1,879,489 markers). Sample quality control was achieved following well-established protocols for genetic case-control association studies [20]. Briefly, discordant sex samples, elevated missing genotypes rate (≥0.03) or outlying heterozygosity rate (>5 SD) were excluded. Principal component analysis was applied to each dataset to remove ancestry outliers with the SMARTPCA software [21]. Further cryptic relatedness was removed by identity by descent (IBD) estimation with the PLINK software randomly eliminating one of each pair of samples having IBD estimates >0.1875 [22]. Samples with an amount of CNV calls larger than 3 SD above the mean were considered CNV-outliers and subsequently excluded from the study [23]. In sum, a total of 790 samples were removed due to QC procedures (POPGEN = 317, SHIP-2 = 314, and ANCORA = 159, Supplementary Fig. S1).

### CNV Detection

Raw intensity CEL files were imported into the Affymetrix Axiom™ CNV Summary Tools Software for allele intensity Log 2 Ratio (Log2R) and B Allele Frequency (BAF) calculations. For SHIP-2 data the Birdsuit algorithm implemented in the Affymetrix Genotyping Console version 4.2 was used [24]. Log2R and BAF values were subsequently introduced into the Nexus Copy Number™ software version 7.5 (BioDiscovery, CA, USA) to make CNV calls using the SNP-FASST2 Segmentation Algorithm and array-specific waviness correction to avoid batch effects. To ensure reliable calls [23, 25] only CNV calls larger than 100 kb and with at least 50 probes were considered for further analysis [24, 26]. Next, since the number of probes of the Affymetrix 6.0 array was more than twice the amount found in the Affymetrix AXIOM array, a 100 probes threshold was applied to the SHIP-2 dataset. Calls overlapping any signal artifact reported in the “Black List” of the ENCODE project (e.g., centromeric) were eliminated [26]. All annotations refer to the genome build GRCh37/hg19. Physical location and sample level annotation for each of the CNVs detected are provided in Supplementary Table S1 at https://github.com/edoper/GSD-CNV. CNV calls in VCF format are publicly available at the European Variation Archive (https://www.ebi.ac.uk/eva/) under the project accession number: PRJEB33136. Dataset specific CNV calls can be accessed for POPGEN analysis: ERZ990179, SHIP analysis: ERZ990178, and ANCORA analysis: ERZ990177.

### Burden analysis

CNV burden analysis was performed by comparing the number of cases and controls with at least one CNV overlapping a member of a gene set of interest, as described [27, 28]. To avoid bias, samples were counted only once, irrespectively of the number of CNVs found. According to the physiopathological knowledge of GSD [1], four gene sets of interest were *a priori* defined using the Gene Ontology [29] database: (i) Lipid Metabolic Process (GO:0006629, *n* = 1,560 genes); (ii) Inflammatory Response (GO:0006954, *n* = 611 genes); (iii) Response to Insulin (GO:0032868, *n* = 290 genes); and (iv) Glucose Metabolic Process (GO:0006006, *n* = 217 genes). Three additional gene sets extracted from the Human Protein Atlas [30] were interrogated according to gene expression in GSD-related tissues (enterohepatic axis): (v) Gallbladder (*n* = 3,788 genes); (vi) Liver (*n* = 3,257 genes); and (vii) Small Intestine (*n* = 3,824 genes). Finally, (viii) a random gene set (*n* = 4,696 genes) [23] was included as a negative control. Complete set of genes interrogated in the burden analysis are listed in Supplementary Table S2 available at https://github.com/edoper/GSD-CNV.

### Statistics analysis

For the burden analysis, the *p*-values, odds ratios (ORs), and 95% confidence intervals (CIs) were calculated with a two-sided Fisher´s exact test with R Statistical Software using Bonferroni correction to adjust for multiple testing [31] (Bonferroni alpha threshold = 0.0055). Generalized linear model (GLM) was carried out with the R statistical software following a binomial model distribution with two possible outcomes (i.e., Case or Control). Five variables were considered to fit the model: CNVs, age, sex, BMI, and dataset. Two-sided *p*-values, ORs, and 95% confidence intervals (CIs) were calculated from corresponding *z*-values and estimates obtained in each model. The Bonferroni alpha threshold was set to 0.0033 considering the 15 models.

## Results

After QC procedures, we included a total of 4778 samples with 1929 GSD cases and 2849 controls (Table 1) in our analysis. QC procedures, CNV detection, and CNV burden analysis are presented in Supplementary Fig. S1. We identified 2936 large and rare CNV events distributed in European and Latin American cohorts (Supplementary Table S1): POPGEN: 821 CNVs (625 Gains and 196 losses); SHIP-2: 1168 CNVs (849 gains and 319 losses); and ANCORA: 947 CNVs (730 gains and 217 losses). As expected, the three cohorts showed consistently more gains than losses [25] (74.18 vs. 25.82%, respectively; Supplementary Fig. S2). We focused on CNVs larger than 100 kb since larger CNVs are more likely to be associated with adverse phenotypes and less prone to false positives [32, 33]. The average size of detected CNVs was between 250 and 500 kb (POPGEN = 41.87%; SHIP-2 = 26.22%; ANCORA = 28.60%). A large proportion of singleton events was observed (POPGEN = 98.23%; SHIP-2 = 81.05%; ANCORA = 87.36%, Supplementary Fig. S2). Since no CNV was found to have a frequency above 1% in their corresponding cohort, all CNVs were considered rare.

**Table 1:** Overview of GSD datasets and patients included in this work

	POPGEN			SHIP-2			ANCORA		
	Cases	Controls	Total	Cases	Controls	Total	Cases	Controls	Total
Samples	1052	905	**1957**	355	1390	**1745**	522	554	**1076**
Sex (% women)	61.5%	42.2%	**52.60%**	64.5%	49.1%	**52.3%**	92.3%	92.4%	**92.40%**
Age (years)	45.7 ± 12.3	59.3 ± 12.8	**52.1** **±** **14.3**	48.4 ± 9.8	47.8 ± 12.1	**47.9** **±** **11.7**	51.3 ± 10.7	49.8 ± 9.6	**50.5** **±** **10.1**
Body mass index	27.6 ± 5.3	26.5 ± 4.1	**27.1** **±** **4.8**	28.6 ± 5.0	26.7 ± 4.2	**27.1** **±** **4.5**	29.4 ± 4.3	28.6 ± 3.9	**29.0** **±** **4.1**
T2D (% affected)	7.1%^a^	6.4%^a^	**6.5%**^a^	NA	NA	**10.9%**^b^	0%	0%	**0%**
CNVs per Sample	0.48	0.35	**0.42**	0.74	0.65	**0.67**	0.88	0.88	**0.88**
CNVs total	500	321	**821**	264	904	**1168**	458	489	**947**

The values given in bold highlight the TOTAL column, which is the value considering all datasets together

All quantitative measures are shown as average ± standard deviation

*T2D* type 2 diabetes, *NA* not available

^a^T2D status in POPGEN dataset is available for 50% of the samples (*n* = 979)

^b^Extracted from Schipf et al.

**Fig. 1:**
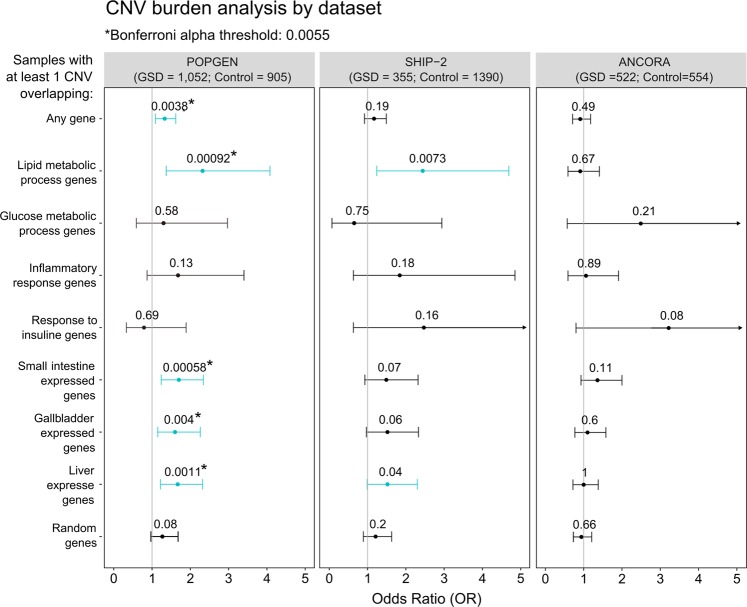
Burden analysis. Forest plot for the three cohorts analyzed (left POPGEN, middle SHIP-2, right ANCORA), the odds ratio (OR, dots) and their respective confidence interval (lines) observed in the gene sets tested: any gene, lipid metabolic process genes, inflammatory response genes, response to insulin genes, glucose metabolic process genes, gallbladder-expressed genes, liver-expressed genes, small intestine-expressed genes and random genes. Adjusted *p* values are shown above the OR, with significant enrichment shown with *

CNV burden analysis was carried out in each cohort separately. To this end, we defined eight specific genes-sets of interest according to either the physiopathology of this complex metabolic disease or gene expression in the enterohepatic axis. We performed a CNV burden analysis and tested if GSD patients were enriched with CNVs in any of the gene sets interrogated (see “Materials and methods” section). First, in the POPGEN cohort, we found a significant association in GSD cases carrying at least one CNV (*p*-value = 3.77 × 10^–3^, OR = 1.33, CI = 1.09–1.62), and this enrichment became stronger when cases had at least one CNV overlapping lipid metabolic process genes (GO:0006629; *p*-value *=* 9.22 × 10^–4^, OR = 2.32, CI = 1.37–4.08) (Fig. 1). CNVs overlapping disease-related tissues were also significant in the POPGEN dataset with CNVs overlapping genes expressed in the small intestine having the strongest signal (p-value = 5.83 × 10^–4^; OR = 1.7; C.I. = 1.24–2.34). Second, in the SHIP-2 cohort we observed nominal association in cases with CNVs overlapping lipid metabolic process genes (p-value = 7.31 × 10^–3^; OR = 2.44; CI = 1.24–4.69), and a clear trend was also observed in tissues of the enterohepatic circuit. Both SHIP-2 signals were no significant after multiple testing correction. On all the gene sets tested, no significant association was observed in the ANCORA cohort (Fig. 1).

**Fig. 2:**
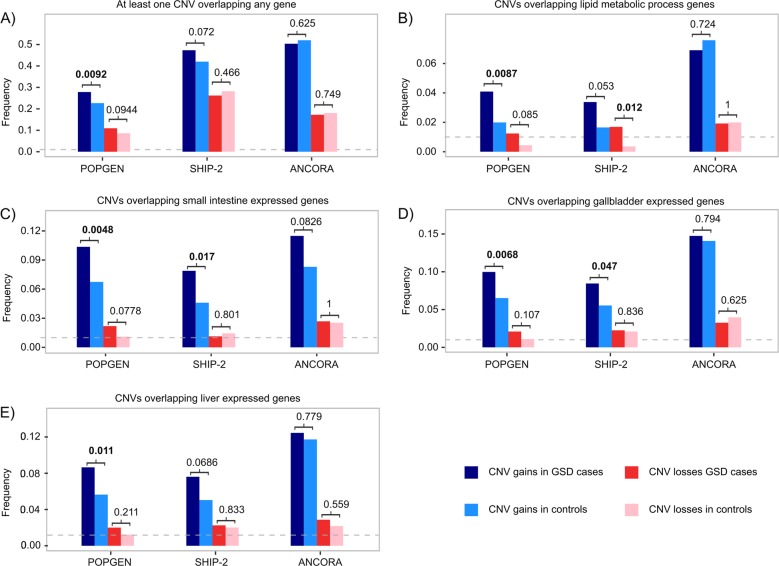
Burden signals by CNV type. Gain and loss frequencies observed in cases and controls in each cohort (POPGEN, SHIP-2, and ANCORA) are shown for **a** samples with at least one CNV; **b** CNVs overlapping lipid metabolic process genes; **c** CNVs overlapping small intestine-expressed genes; **d** CNVs overlapping gallbladder-expressed genes; **e** CNVs overlapping liver-expressed genes. Blue bars: CNV gain frequency in cases; light blue bars: CNV gain frequency in controls; red bars: CNV loss frequency in cases; pink bars: CNV loss frequency in controls. If the difference between the frequencies observed in cases and controls is significant (*p*-value < 0.05), the *p*-value is shown above the corresponding bars. For comparison, the broken horizontal line marks 0.01 frequencies

To unveil whether a specific type of CNVs drives the observed associations, we conducted burden analysis considering gains and losses separately. Regarding gains (Fig. 2, dark and light blue bars), we observed enrichment in the POPGEN cohort, namely in samples with: at least one CNV (*p*-value = 9.23 × 10^–3^; OR = 1.32; CI = 1.07–1.63), CNVs overlapping lipid metabolic process genes (*p*-value = 8.70 × 10^–3^; OR = 2.1; CI = 1.18–3.9), CNVs overlapping small intestine genes (*p*-value = 4.78 × 10^–3^; OR = 1.6; CI = 1.14–2.26) and CNVs overlapping gallbladder expressed genes (*p*-value = 6.80 × 10^–3^; OR = 1.59; CI = 1.13–2.26). In the POPGEN cohort, all the CNV associations with GSD were driven by gains with no significant differences observed between losses found in cases and controls (Fig. 2, dark and light red bars). Same pattern was observed in the SHIP-2 dataset which showed association with GSD in CNVs overlapping lipid metabolic process genes (*p*-value = 0.01; OR = 2.08; CI = 0.93–4.4), CNVs overlapping gallbladder expressed genes (*p*-value = 0.05; OR = 1.57; CI = 0.98–2.48) and CNVs overlapping small intestine expressed genes (*p*-value = 0.02; OR = 1.77; CI = 1.08–2.86). With the exception of CNVs overlapping lipid metabolic process genes, all associations in the SHIP-2 dataset were driven by gains (Fig. 2). No significant association in gains and losses was observed in the ANCORA dataset.

**Fig. 3:**
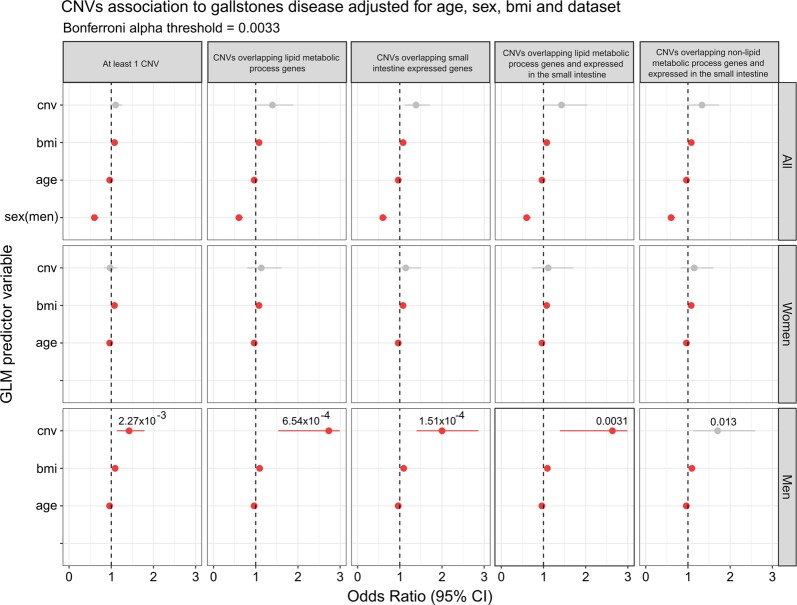
CNVs’ association to gallstones disease adjusted for age, sex, BMI and dataset. Forest plots of odds ratios obtained for each of the predictors analyzed by GLMs in all individuals (first row) and stratified by sex (second and third row, respectively). GLMs are shown for at least one CNV (first column); CNVs overlapping lipid metabolic process genes (second column); CNVs overlapping small intestine-expressed genes (third column); CNVs overlapping lipid metabolic process genes and expressed in the small intestine (fourth column), and CNVs overlapping non-lipid metabolic process genes and expressed in the small intestine (fifth column). In total, 15 GLMs were carried out. Circles alongside horizontal lines denote predictor OR and 95% CI, respectively. For each forest plot the corresponding OR scale is shown on the horizontal axis. Red circles denote significant *p*-values (*p*-value < 0.05)

Next, to increase the statistical power and extract general conclusions about the observed associations, we performed a general linear model adjusting for sex, age, BMI, and dataset. We tested for CNV association to GSD using five sets of potential predictors: (1) at least one CNV, (2) CNVs overlapping lipid metabolic process genes, (3) CNVs overlapping small intestine expressed genes, (4) CNVs overlapping lipid metabolic process genes and expressed in the small intestine, and (5) CNVs overlapping nonlipid metabolic process genes and expressed in the small intestine (Fig. 3). Overall, our results indicate that when samples are considered together and adjusted, CNVs do not show significant association with GSD (Fig. 3, first row). As expected, age (*p*-value = 5.31 × 10^–41^; OR = 0.96; CI 95% = 0.95–0.97), BMI (*p*-value = 1.42 × 10^–23^; OR = 1.07; CI 95% = 1.06–1.09) and sex (*p*-value = 9.20 × 10^–12^; OR = 0.6; CI 95% = 0.51–0.69) were significantly associated to GD, yet with a small effect size on our sample set. Since the sex variable showed the strongest effect with a clear protective outcome for men we stratified the GLM models by sex. Sex-stratified GLMs retained association of the age and BMI variables and revealed no CNV signal in women (Fig. 3, second row). In contrast, CNVs were significantly associated with GSD in men when considering samples with at least one CNV (*p*-value = 2.27 × 10^–3^; OR = 1.42; CI 95% = 1.13–1.78). The strongest effect was observed for CNVs overlapping lipid metabolic genes (*p*-value = 6.54 × 10^–4^; OR = 2.76; CI 95% = 1.53–4.89) followed by CNV overlapping small intestine expressed genes (*p*-value = 1.51 × 10^–4^; OR = 2.00; CI 95% = 1.39–2.86). Since genes related to lipid metabolic process can also be expressed in the small intestine, we therefore examined if these signals were independent of each other. We found that the signal remained significant when considering CNV overlapping lipid metabolic process genes and expressed in the small intestine (*p*-value = 3.1 × 10^–3^; OR = 2.6; CI 95% = 1.38–5.04). Interestingly, when removing lipid metabolic process genes from the small intestine expressed genes a nominal signal is still observed (*p*-value = 0.0133; OR = 1.70; CI 95% = 1.11–2.59).

Finally, to gain insights from a gene-centric perspective, we applied a series of filtering criteria to extract candidate genes with potential involvement in the development of GSD through a CNV mechanism. From the list of 1560 genes associated with the Lipid metabolic process (GO:0006629), we found 369 expressed in the small intestine. Among these, 51 genes were overlapped by a CNV event in at least one GSD patient. Finally, by removing those genes overlapped by at least one CNV from the control group we were able to identify 23 candidate genes that were: (i) exclusively affected by CNVs in GSD patients, (ii) involved in lipid metabolic process and (iii) expressed in the small intestine (Table 2). These genes displayed more direct interactions than expected (PPI enrichment *p*-value: 0.0025) and in addition to lipid metabolic process they showed enrichment for drug catabolic process (GO:0042737; fold enrichment = 31.99; adjusted *p*-value = 4.24 × 10^–03^), driven by the *POR*, *BDH1*, *OXCT1*, *PCK1*, and *SULT2A1* genes, and alcohol metabolic process (GO:0006066; fold enrichment = 21.78; adjusted *p*-value = 1.99 × 10^–04^), driven by the *APOL2*, *APOL1*, *HNF1A*, *SULT2A1*, *PCK1*, *PRKAA1*, and *OCRL* genes (Supplementary Fig. S3) [34].

**Table 2:** CNV candidate genes for GSD

Gene ID	N° GSD cases	N° controls	Case sex	Chr	CNV start (hg19)	CNV end (hg19)	Type	Cohort
ALAS1	1	0	Male	3	52,082,780	53,128,685	Loss	POPGEN
BDH1	1	0	Female	3	196979974	197340846	Gain	SHIP-2
AGPAT9	1	0	Female	4	84,039,517	85,091,231	Gain	ANCORA
PRKAA1, OXCT1	1	0	Female	5	37,623,393	42,403,769	Gain	ANCORA
POR	1	0	Male	7	75,220,393	75,956,150	Gain	POPGEN
AGPAT5	1	0	Male	8	6,254,942	6,661,675	Gain	POPGEN
LYN	1	0	Male	8	56,753,726	57,051,296	Gain	POPGEN
PLIN2	1	0	Female	9	19,031,461	19,226,776	Gain	POPGEN
PDHX	1	0	Male	11	34,592,852	34,952,574	Gain	POPGEN
GOLT1B	1	0	Female	12	20837585	21981183	Gain	SHIP-2
HNF1A	1	0	Female	12	121,202,411	121,744,337	Gain	POPGEN
RB1	1	0	Male	13	48646292	49269685	Loss	SHIP-2
FECH, ATP8B1	2	0	Male, Female	18	55,206,938	55,465,080	Gain	POPGEN
SULT2A1	1	0	Female	19	48,085,947	48,392,233	Gain	POPGEN
RBL1	1	0	Female	20	35524811	35731498	Gain	SHIP-2
PCK1	1	0	Male	20	55,665,634	56,143,223	Gain	POPGEN
APOL1, APOL2	1	0	Male	22	36,556,147	36,749,312	Gain	POPGEN
TBL1X	1	0	Female	X	9,443,988	9,763,908	Gain	ANCORA
PDHA1	1	0	Male	X	18,662,632	19,449,407	Gain	POPGEN
OCRL	1	0	Female	X	113,759,647	150,222,670	Loss	POPGEN

## Discussion

We report for the first time a significant enrichment of CNV burden in men with GSD. The observed signal was characterized by two main components: first, the association was sex specific, affecting men exclusively with no apparent association in women; and second, the association was highly heterogeneous in composition, suggesting that the observed results arise as a cumulative burden of different CNVs in different genes.

In the hypothesis free approach (i.e., samples having at least one CNV) we evaluated if the burden of samples with at least one CNV was higher in patients than in controls. As shown in Fig. 3, this holds true only for CNVs observed in men. This observation does not arise from sex-specific effects. When we tested sex as a potential predictor of having at least one CNV we observe no significant difference between women and men (*p*-value = 0.46; OR = 1.05; CI = 0.92–1.19). Hypothesis free CNV signal in men allow us to conclude that there is a significant enrichment of men with GSD that have large and rare CNVs compared with controls, however the signal does not point out to any specific variant or region in the genome. The introduction of hypothesis-driven gene sets allowed us to identify stronger CNV associations and narrow down the signal to specific regions of the genome (i.e., CNVs overlapping lipid metabolic process genes). Our approach was focused on large and rare CNV events that on average were found less than one time per sample (Table 1). While testing for more or even all possible gene sets could provide further specificity to the CNV signal, these signals most likely will not survive multiple testing correction. Larger cohorts and next generation sequencing platforms will provide further power and sensitivity to the CNV analysis.

Overall, the observed CNV enrichment was mostly driven by gains. Considering that the majority of these types of variants ranged between 250 and 500 kb in size, the most likely mechanism by which CNVs increase the risk for GSD would be a gain of function of the affected genes due to the increase of complete gene copies. We note that losses also contributed to the association, although to a lesser degree (Fig. 2) and that, 3 out of 23 genes identified as candidate genes in the present study (*ALAS1*, *RB1*, and *OCRL*) had a unique loss event, which was not observed in controls (Table 2). In this regard, both gain- and loss-of-function mechanisms have been related to the complex etiology of GSD. For example, common SNP variants associated with GSD cause gain-of-function of ABCG5/8 leading to cholesterol hypersecretion [35], as well as a loss-of-function of UGT1A1, which leads to saturation of unconjugated bilirubin in the gallbladder [7].

CNV events with the highest risk for GSD in men were the ones overlapping genes involved in lipid metabolic process and expressed in the small intestine (OR = 2.73). An additional nominal signal was observed independent of lipid metabolic process in the small intestine, suggesting that additional pathways conferring risk to GSD through CNVs may be identified in larger, future studies.

Initially, CNV burden was interrogated independently for each available dataset, given their evident differences in ethnicity (European vs Admixed Latin American) and genotyping platform (Affymetrix Axiom vs Affymetrix 6.0). At this stage, we observed direct replication of results within the European cohorts of POPGEN and SHIP-2, in particular regarding CNVs overlapping lipid metabolic process genes and genes expressed in disease-related tissues. The smallest dataset ANCORA did not reach nominal significance individually. In this regard, Population’s specific effects have been well established for GSD [1], especially between populations of European and admixed Amerindian origin [2]. However, given the uneven sex composition of our Chilean dataset [6], we suspect that the inability to reach independent significance was due to the small number of men with GSD (*n* = 40) in the sample rather than the presence of populations specifics effects. Along the same line, the burden was focused on large and rare CNV events, with >80% of calls observed only once in Europeans and/or Latin American datasets. The low frequency of detected CNV events suggest that they are not frequently established in any specific population and that the bias introduced by comparison of populations with different ethnicities should be minor. Familial GSD studies allowing the detection of de novo CNV events will be necessary to confirm the origin of associated CNVs. Our results were obtained using a generalized linear regression model that allowed us to adjust for known co-founders in GSD, namely age, BMI, sex, and dataset. T2D status was not possible to include in the model since it was not available for 50% of POPGEN samples and entirely unavailable for SHIP-2 samples. Still, based on partial values shown in Table 1 T2D samples represent <10% of the whole dataset (0% for ANCORA, ~6.5% for POPGEN and ~10.9% for SHIP-2 data).

Our results showed a clear sex-specific effect towards CNVs overlapping lipid metabolic process genes. In this context, sex-specifics effects are commonly found in a wide spectrum of common and complex diseases including cardiovascular [27], neuropsychiatric [27], and metabolic phenotypes [36], which are strongly influenced by sex hormones. For GSD, these effects have already been reported and the disease is known to be more prevalent in women than men [1]. Still, male-specific associations have been found inside the gene *UDP glucuronosyltransferase family 1 member A1*, UGT1A1 (rs6742078) [7].

The observed associations could not be attributed to one CNV nor to an specific gene and thus CNVs may confer risk to GSD through multiple highly rare and penetrants events. In an effort to identify genes with the strongest potential to be involved in the development of GSD by a CNV-related mechanism, we highlight 23 genes involved in lipid metabolic process, which are expressed in the small intestine and exclusively affected by CNV in cases. Direct functional links between the candidate genes and the etiology of GSD is available for: the lipid transporter ATPase phospholipid transporting 8B1, (*ATP8B1*) gene [28], the RB transcriptional corepressor 1, (*RB1*) gene [37], the hepatocyte nuclear factor 1α (*HNF1A*) gene [38], the bile salt transporter gene sulfotransferase family 2 A member 1, (*SULT2A1*) gene [10], and the Apolipoprotein L1 and L2 (*APOL1* and *APOL2*) involved in lipid transport. In addition, we note that the 23 candidate genes were enriched with 7 genes extremely intolerant to loss-of-function genetic variation (i.e., *ALAS1, HNF1A, LYN, OCRL, PDHA1, RB1*, and *TBL1X;* hypergeometric test *p*-value = 0.033) and variants within these genes are rarely found in the general population [39]. We hypothesize that these genes are strong candidates for future functional studies.

As suggested by CNVs overlapping nonlipid metabolic process genes that are expressed in the small intestine (Fig. 3) and the drug catabolism and alcohol metabolism signals (Supplementary Fig. S3), additional nonlipid candidate genes remain to be discovered. In this regard, a study on 62,166 patients with diabetes mellitus II, a relevant risk factor for GSD development, identified 167 genome-wide common signals that only explained between 2.2 and 6.7% of disease heritability [40]. This observation is in agreement with the ‘Common Disease, Rare Variant (CDRV)’ model, that argues that cumulative rare and highly penetrant variants could explain most of the susceptibility to common diseases [41]. Our results supports a CDRV model on GSD and provide evidence that multiple rare and large CNVs can play a major role in the pathogenesis.

In summary, our study provides the first evidence of association of CNVs with GSD and will contribute to better understand the etiology of this complex and common metabolic disorder.

## Supplementary information

Supplementary Material
