# Supplementary material for: Copy number variants in lipid metabolism genes are associated with gallstones disease in men
Source: Eur J Hum Genet. 2019 Sep 4;28(2):264–73. doi: 10.1038/s41431-019-0501-7 (PMC6974590; doi:10.1038/s41431-019-0501-7)
Supplement: Supplementary file 1 — Supplementary Material [file 41431_2019_501_MOESM1_ESM.docx]

**Copy number variants in lipid metabolism genes are associated with gallstones disease in men**

# Supplementary Information

**Supplementary Figure S1.**


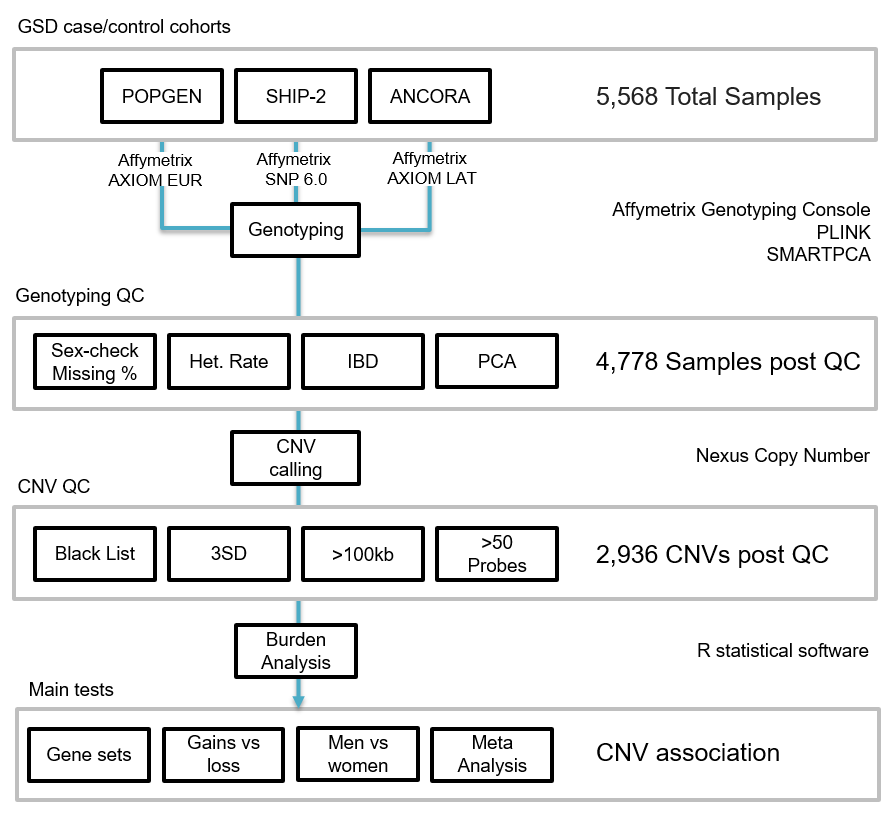


**Study workflow.** The study includes 5,568 initial samples belonging to three gallstones disease cohorts: POPGEN, SHIP-2 and ANCORA. Genotyping was performed following standard quality controls (QCs) for genome wide association studies, including missingness percentage, principal component analyses (PCA), identity by descent (IBD) and heterozygosity rates were used for sample and SNP filtering (See Methods). Nexus Copy Number software was used as the primary CNV calling algorithm. For CNV QC procedures, CNVs in known repetitive regions ENCODE “Black list”, either above 3SD from the media, smaller than 100kb or with less than 50 probes involved were not considered. Finally, burden analyses were performed considering 4,778 individuals with 2,936 CNVs detected.

**Supplementary Figure S2.**

**
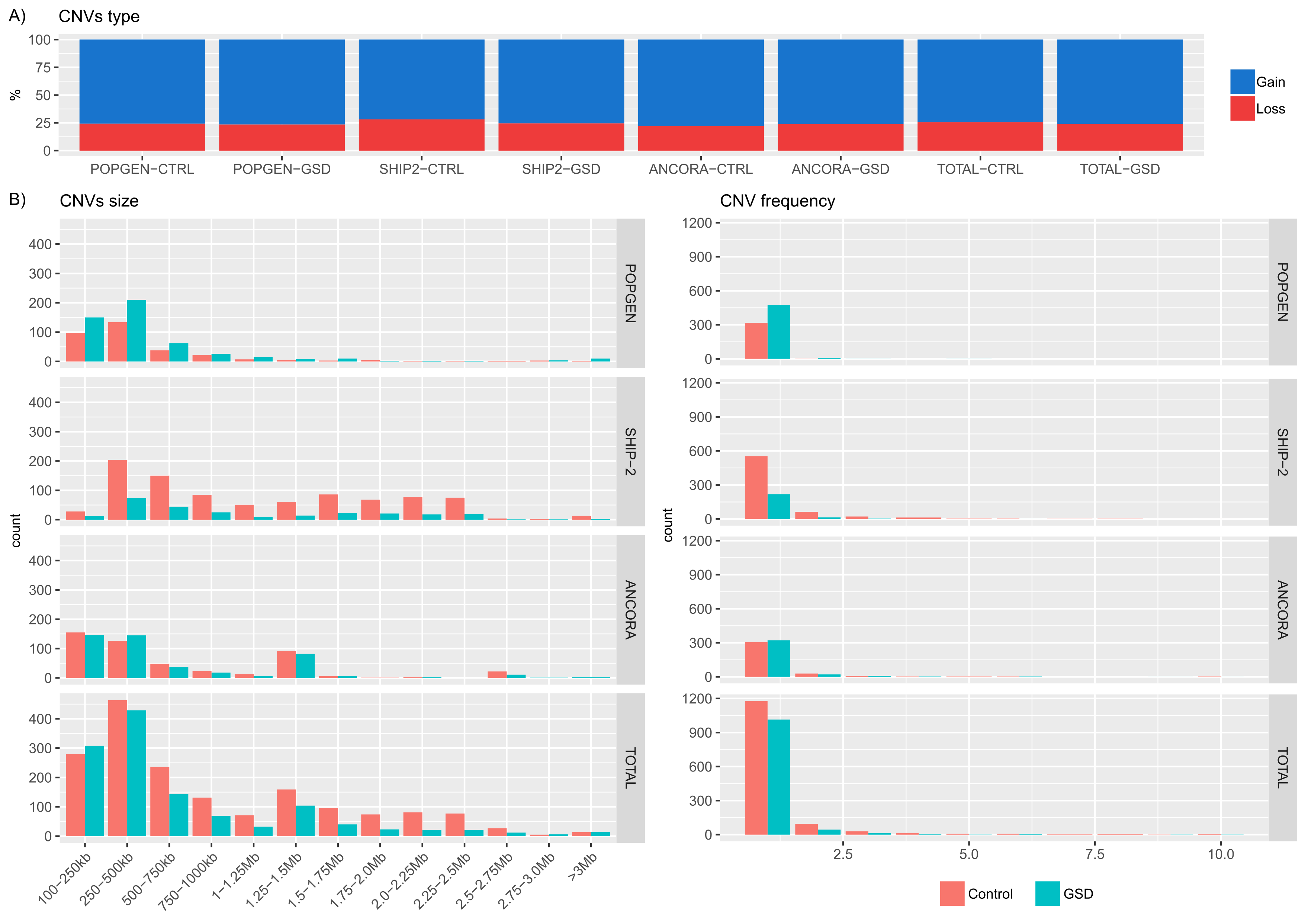
**

**CNV type, size and frequency distribution.** **A)** CNVs gain (blue bars) and losses (red bars) frequency distribution observed in each dataset (POPGEN, SHIP-2 and ANCORA) and in combination (TOTAL). Cases and control frequencies are shown separately. **B)** CNV size (left panel) and frequency (right panel) distribution by intervals of 250 kb and by number of individuals respectively is shown for each dataset (POPGEN, SHIP-2 and ANCORA) and in combination (TOTAL). Frequencies in cases are shown in light red bars while frequencies in controls are shown in light green bars.

**Supplementary Figure S3**


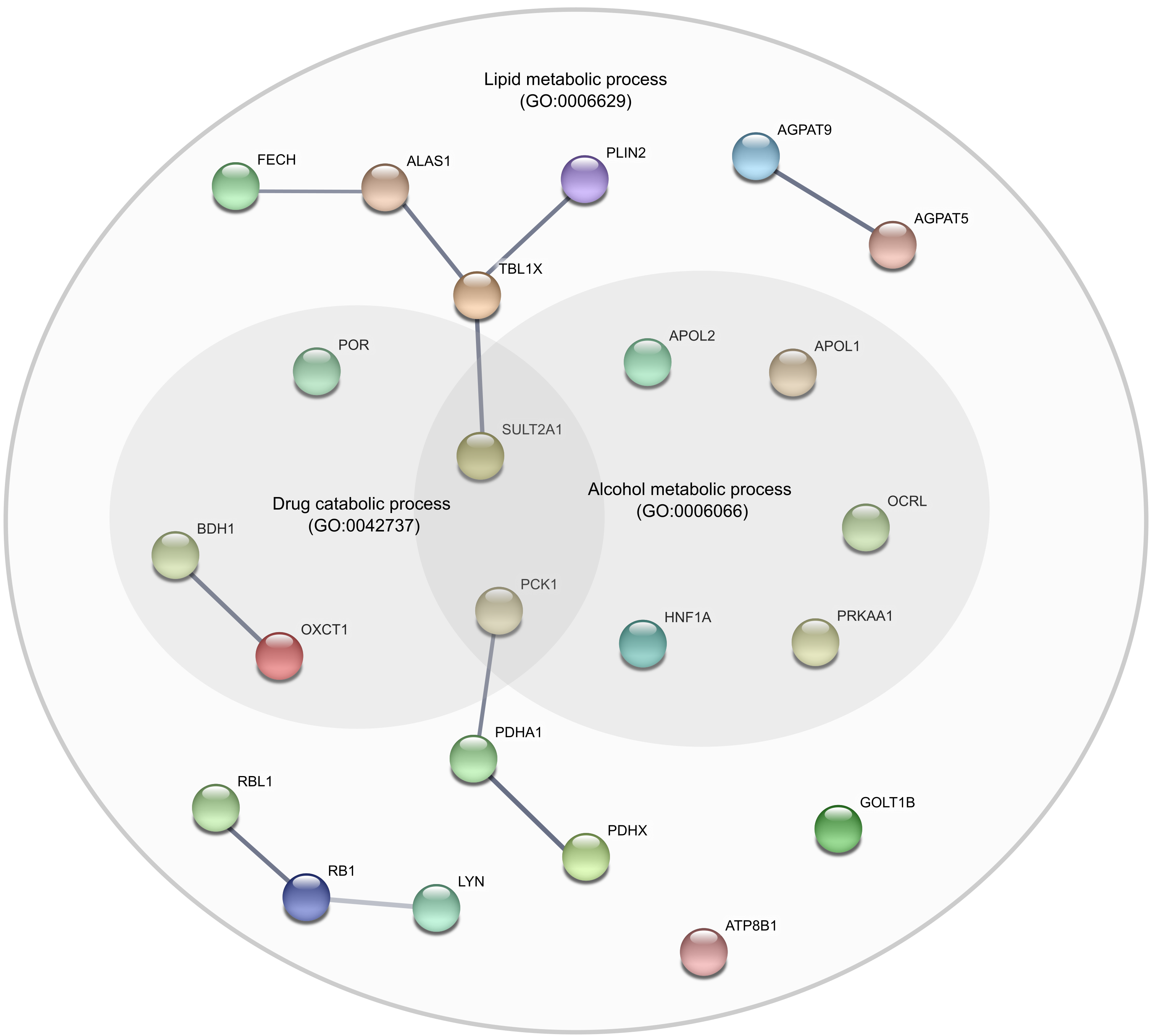


**Network analysis results.** The 23 candidate genes are displayed as nodes alongside known interactions between them (grey lines) according to STRING v10 database. All candidates genes belongs to the Lipid metabolic process category (GO:006629). Gene ontology enrichment analysis showed additional enrichment for Drug catabolic process (GO:0042737) and Alcohol metabolic process (GO:006066). Genes contained in each category are shown inside corresponding grey bubbles.
